# Editorial: The youth elite football players

**DOI:** 10.3389/fspor.2024.1399143

**Published:** 2024-03-22

**Authors:** Thomas Rostgaard Andersen, James J. Malone, Morten Bredsgaard Randers

**Affiliations:** ^1^ Research Unit of Sport and Health Science, Department of Sports Science and Clinical Biomechanics, Faculty of Health Sciences, University of Southern Denmark, Odense, Denmark; ^2^ School of Health Sciences, Liverpool Hope University, Liverpool, United Kingdom

**Keywords:** injury risk, mental health, match performance, recovery, player development, psychological and social factors

**Editorial on the Research Topic** The youth elite football players

Youth football player development involves a delicate interaction between football-specific technical, tactical, physical, and mental aspects, as well as various biological, sociological, and cultural conditions. In recent years, this has attracted intensified academic interest. This research topic dedicated to female and male elite youth football players, presents 10 original research papers related to injury risk and mental health, match performance and recovery, player development and talent identification as well as, psychological and social factors.

Improving player well-being and mitigating injury risks in youth football is paramount in preserving long-term development. Two studies by Andersen and colleagues provide insights into these areas, shedding light on the challenges faced by players and the strategies needed to address these effectively.

The first study reveals the load distribution and injury burden in male youth elite players over a full competitive season. Hip/groin injuries emerge as a prominent concern, while knee injuries impose the greatest burden and the most playing time lost. These findings underscore the urgent need for targeted injury prevention measures and comprehensive player management strategies.

The second study addresses the relationship between load exposure, wellness, and psychological variables among male and female youth national team players. It highlights the impact of international competitions on players' physical and mental well-being, with increased stress levels observed during periods of international competitions. Importantly, the study identifies key correlations between stress, load, and well-being, emphasizing the interconnected nature of these factors.

Together, these studies underscore the multifaceted nature of player health and performance in youth football. By leveraging data-driven approaches and implementing evidence-based interventions, stakeholders may effectively safeguard the well-being of young athletes and nurture their potential on and off the field.

Expanding on player performance and recovery, understanding the impact of match demands and recovery intervals is pivotal for optimizing performance. The study by Franceschi et al. examines the reliability and sensitivity to change post-match physical performance measures in elite youth football players. Notably, isometric posterior chain peak force and torque, along with various CMJ parameters, emerged as robust indicators for monitoring neuromuscular performance. These findings underscore the importance of employing precise assessment protocols to track performance variations and inform targeted recovery interventions. Furthermore, the study by Wilke et al. addresses differences between 48-h and 72-h rest intervals following international tournament matches. While no significant differences were observed in performance or perceptual parameters between the two rest intervals, biochemical markers of inflammation and muscle damage were markedly elevated after matches with shorter rest periods. This highlights the physiological strain associated with compressed recovery timelines and underscores the necessity of adequate rest for player recuperation and injury prevention. Collectively, these studies offer valuable insights into the interplay between match demands, recovery dynamics, and player performance in youth elite football.

Player development and talent identification, requires a nuanced understanding of the factors shaping player fitness, organizational structures, and stakeholder coherence. Three studies shed light on critical aspects of youth elite football development, offering valuable insights for coaches, administrators, and policymakers.

The first study (Gonaus et al.) investigates the decade-long trends in the fitness of elite Austrian youth football players, employing propensity score matching to control for confounding variables. Results indicate notable improvements in speed-related attributes among recent players, highlighting the evolving demands of modern football. However, declines in flexibility and upper-limb power underscore the need for comprehensive training programs that address diverse physical attributes.

A comprehensive survey of operational practices in youth elite football academies and national federations worldwide is presented in the study by Gregson et al. Findings reveal strategic alignment and shared decision-making within medical and performance units, alongside widespread research, and development activities. These insights offer valuable implications for optimizing player development programs and enhancing collaboration across organizational units. Sweeney et al. examines stakeholder coherence throughout the Irish football player pathway, revealing significant gaps in understanding and implementation of developmental principles. While stakeholders demonstrate alignment with talent development principles, inconsistencies in strategic aims and relationships underscore the need for organizational intervention and structural change. Recommendations are provided for fostering stakeholder coherence and aligning policies with long-term player development goals. Together, these studies provide a comprehensive overview of elite youth football development, emphasizing the importance of continuous monitoring, collaboration, and adaptation to meet the evolving demands of modern football. By integrating these insights into practice, stakeholders can enhance player development pathways and nurture the next generation of football talent effectively.

Finally, three studies address achievement, psychological, and social factors in football. For elite male players, release from professional academies may result in psychological distress, with an urgent need for robust support structures to aid players through this transition (McGlinchey et al.). In elite women's football, the decision to persevere or depart is influenced by a myriad of factors, from financial constraints to divergent motivations. While steps have been taken to improve conditions, tailored interventions are necessary to sustain and empower female athletes in their professional pursuits (Bjerksæter & Lagestad). Finally, the interplay between relative age effect and personality constructs underscores the importance of holistic development in youth football. Younger players display higher self-confidence, while older counterparts exhibit greater team orientation. This highlights the need to cultivate well-rounded individuals who can overcome physical disparities through psychological resilience (Bolckmans et al.).

Altogether, the research topic articles underscore the multifaceted nature of youth football player development, addressing critical aspects such as injury prevention, match performance, talent identification, and psychological well-being. Moreover, the studies emphasize the importance of continuous monitoring, evidence-based interventions, and tailored support structures to nurture the next generation of football talent effectively. By integrating these insights into practice, stakeholders may navigate effectively fostering holistic growth, resilience an improved performances among female and male youth elite football players.

